# Influenza viral vectors expressing two kinds of HA proteins for bivalent vaccines against clade 2.3.4.4 and clade 2.3.2.1 H5 HPAIVs

**DOI:** 10.1038/s41598-018-27722-5

**Published:** 2018-06-19

**Authors:** Guangyu Hou, Jinping Li, Yan Wang, Suchun Wang, Cheng Peng, Xiaohui Yu, Jihui Jin, Wenming Jiang

**Affiliations:** 1 China Animal Health and Epidemiology Center, Qingdao, China; 2 Shanghai Entry-Exit Inspection and Quarantine Bureau, Shanghai, China

## Abstract

The H5 highly pathogenic avian influenza viruses (HPAIVs) in China pose a serious challenge to public health and the poultry industry. In this study, we constructed a replication-competent recombinant influenza A virus of clade 2.3.4.4 Н5N1 expressing the clade 2.3.2.1 H5 HA1 protein from a tricistronic NS segment. We used a truncated NS1 protein of 73 amino acids combined with a heterologous dimerization domain to increase protein stability. H5 HA1 and nuclear export information were fused in frame with a truncated NS1 open reading frame, separated by 2A self-processing sites. The resulting PR8-H5-NS1(73)H5 stably expressed clade 2.3.4.4 H5 HA and clade 2.3.2.1 H5 HA1 proteins and exhibited similar *in vitro* growth kinetics as the parental PR8-2344H5 virus. PR8-H5-NS1(73)H5 induced specific hemagglutination-inhibition (HI) antibody against clade 2.3.4.4 H5 that was comparable to that of the combination vaccine of PR8-2344H5 and PR8-2321H5. HI antibody titers were significantly lower against clade 2.3.2.1 H5 virus than with the combination vaccine. PR8-H5-NS1(73)H5 completely protected chickens from both clade 2.3.4.4 and clade 2.3.2.1 H5 HPAIVs challenge. Our results suggested that PR8-H5-NS1(73)H5 was highly immunogenic and efficacious against both clade 2.3.4.4 and clade 2.3.2.1 H5 HPAIVs in chickens.

## Introduction

H5N1 avian influenza viruses (AIVs) have been detected in more than 60 countries and have caused economic losses for the poultry industry worldwide^1^. Of note, the H5N1 AIVs have become enzootic in poultry and wild birds in China (including Hong Kong special administrative region [SAR]), Bangladesh, Eastern India, Indonesia, Vietnam and Egypt^2^. The H5 viruses have evolved into diverse clades and subclades based on the genetic sequences of their HA genes^3^. The clade 2.3.2.1 and clade 2.3.4.4 viruses are cocirculating in wild birds and poultry in several countries, and the clade 7.2 viruses have also been detected in chickens in several provinces in northern China^3^. Molecular epidemiological surveys of AIVs show that the clade 7.2 viruses have not been detected and the clade 2.3.2.1 viruses have decreased significantly since 2015 (data not published). Currently, clade 2.3.4.4 viruses are the dominant epidemic strains. However, some outbreaks are caused by clade 2.3.2.1 H5 viruses (www.oie.int/).

Vaccination is an important strategy to control H5N1 AIVs among poultry in mainland China, Hong Kong SAR, Vietnam, Indonesia, and Egypt^1,4^. The vaccine strains used in China have been updated several times since 2004 to ensure an antigenic match between the vaccines and the prevalent strains^5^. The inactivated H5N1 vaccine produced from vaccine strain Re-8 was generated by reverse genetics and contains the HA and NA genes of a clade 2.3.4.4 virus, A/chicken/Guizhou/4/2013(H5N1). The vaccine has been widely used to control clade 2.3.4.4 AIVs in mainland China and Hong Kong since 2016^6^. Since August 2017, the combination vaccine of H5 Re-8 and H7 Re1 has been used to control highly pathogenic H5N1 and H7N9 AIVs throughout the country.

The influenza A virus contains a segmented genome of eight negative-strand RNA fragments. Of these, the smallest fragment is nonstructural (NS) protein, encoding the two proteins viral NS1 and nuclear export protein (NEP), which is a convenient target for genetic manipulation since NS1 can tolerate foreign sequences exceeding its own length^7^. Thus, a mature HA1 sequence of clade 2.3.2.1 H5N1 virus was inserted into the ORF of NS1. The А/Puerto Rico/8/34 (H1N1) strain was used as the backbone for obtaining influenza A viral vectors expressing H5N1 mature HA1 sequence as fusion protein with the N-terminal 73 amino acid residues of NS1 and expressing HA and NA proteins of clade 2.3.4.4 H5 that replaced the corresponding proteins of PR8 virus. The immunogenicity and protective efficacy in chickens of a bivalent vaccine against challenge with both clade 2.3.4.4 and clade 2.3.2.1 H5 HPAIVs was evaluated.

## Materials and Methods

### Ethics statements and facility

All animal experiments were performed in accordance with regulatory standards and guidelines approved by the Animal Care and Use Committee of China Animal Health and Epidemiology Center, and the approved is NO. CAHEC-2017-007. All experiments with lethal H5 viruses were performed in a biosafety level 3 facility, and all animal experiments were performed in high-efficiency particulate air-filtered isolators at the China Animal Health and Epidemiology Center.

### Viruses and cells

HPAIVs A/chicken/Fujian/5/2016(H5N6) (FJ/5, clade 2.3.4.4) and A/chicken/Jiangsu/7/2017(H5N1) (JS/7, clade 2.3.2.1) were isolated from chickens that died in outbreaks and propagated in 10-day-oldspecific-pathogen-free (SPF) embryonated chicken eggs (ECEs). Their intravenous pathogenicity indexes were 3.00 and 2.86 using ten 6-week-old SPF chickens intravenously inoculated with 0.1 ml 1/10 dilution of fresh infectious allantoic fluid from FJ/5 and JS/7, according to the World Organization for Animal Health manual. The 293 T human embryonic kidney cells (HEK 293 T) were maintained in Dulbecco’s modified Eagle medium supplemented with 10% FCS and kept at 37 °C in 5% CO_2_.

### Construction of plasmid pHW-NS1(73)-H5HA1-NEP

The coding sequence of Dmd/FMDV-2A was generated synthetically (Sangon, Shanghai, China) and cloned into the pcDNA3 vector using the restriction sites *Not*I and *Xba*I. The sequence for the first 73 amino acids of NS1 was amplified by PCR from plasmid pHW198-NS^8^ and cloned 5′ to and in frame with Dmd/FMDV-2A using *Bam*HI and *Esp*EI. NS(73)-Dmd/FMDV-2A was cloned into the pHW2000 plasmid using *Bam*HI and *Mun*I restriction sites.

The mature HA1 coding sequence derived from JS/7 was cloned 3′ of and in frame with the FMDV-2A cleavage site using *B*glII and *Eco*RI. The PTV-1 2A cleavage site was fused to the NEP coding sequence by fusion PCR and cloned 3′ of and in frame with the mature HA1 coding sequence using *Eco*RI and *Bst*EII restriction sites.

### Generation of recombinant viruses

All viruses were generated by a standard reverse genetics method using 8 bidirectional plasmids pHW2000^9^. HEK 293 T cells were co-transfected with 0.8 μg of each of the six pHW-plasmids (pHW191-PB2, pHW192-PB1, pHW193-PA, pHW195-NP, pHW197-M, and pHW-NS1(73)-H5 HA1-NEP), as well as the HA and NA genes of the FJ/5 virus using Lipofectamine 3000 transfection reagent (Life Technologies, Carlsbad, CA, USA). The pHW-plasmids expressing six internal genes were all originated from A/PR/8/34 (H1N1) (PR8) virus. The HA sequence of FJ/5 virus was attenuated by removing the multibasic amino acid motif from RERRRKRG to RETRG in the HA cleavage site. After 24 h, TPCK-treated trypsin (Sigma–Aldrich Corporation, St. Louis, MO, USA) was added to a final concentration of 2 μg/ml. After 72 h, supernatants of transfected cells were collected and used to inoculate10-day-old SPF ECEs incubated at 37 °С for 72 h. Vaccine batches were produced in SPF ECEs after five egg passages of viral constructs.

Recombinant viruses were also rescued using pHW198-NS with the HA protein sequence of FJ/5 (clade 2.3.4.4) or JS/7 (clade 2.3.2.1) virus attenuated by removing the multibasic amino acid motif in the HA cleavage site, which were named PR8-2344H5 and PR8-2321H5, respectively.

### Determination of the 50% embryo infectious dose (EID_50_) of the viruses

Infectious titers of viruses were determined by standard methods using 10-day old SPF ECEs. Viral suspensions (10^−1^ to 10^−9^ dilutions) were prepared in PBS (pH 7.2) and allantoic cavities of five ECEs were infected with 0.1 ml dilution. ECEs were incubated at 37 °C at relative humidity 60 for 72 h. Viral titers were determined by hemagglutination assays. Viral titers were calculated and expressed as log10 EID_50_/ml.

### Growth kinetics *in vitro*

SPF ECEs at 10-days-old were infected with 10^4^ EID_50_ PR8-2344H5, PR8-2321H5, or PR8-H5-NS1(73)H5 virus. Samples of allantoic fluid from five eggs per virus were taken at 0, 4, 8, 12, 24, 36, and 48 h. Viral titers of samples were determined by EID_50_ analysis.

### Genetic stability of PR8-H5-NS1(73)H5 virus

Ten consecutive passages of PR8-H5-NS1(73)H5 virus were conducted in 10-day-old ECEs for genetic stability testing. The allantoic cavity of ECEs was infected using10^4^ EID_50_. Genetic stability of viral constructs was confirmed by reverse transcription polymerase chain reaction (RT-PCR) using NS segment-specific primers (sense 5′-GTA GAT TGC TTT CTT TGG-3′ and antisense5′-CTA AAT AAG CTG AAA CGA-3′). At passages 1, 3, 5 and 10, the size of the NS amplicon was compared to the pHW plasmid encoding the corresponding gene. NS1-fusion proteins encoding genes of viral constructs were sequenced at passages 1, 3, 5 and 10 using the Sanger method with commercial kit Prism BigDye™ Terminator v3.1 (Applied Biosystems, Foster City, CA, USA) on an automatic sequencer Genetic Analyser 3730XL.

### Analysis of H5 HA1 proteins by western blot

Samples of allantoic fluid from uninfected ECEs and ECEs infected with PR8-2344H5, PR8-2321H5, or PR8-H5-NS1(73)H5 virus were purified by ultracentrifugation through a 20% sucrose cushion. 293 T cells were transfected with plasmid pHW-NS1(73)-H5HA1-NEP as a control. Samples were mixed with Laemmli buffer containing β-mercaptoethanol and boiled for 5 min. Proteins were separated using SDS-PAGE, and targeted proteins were visualized by western blot using anti-H5 for clade 2.3.4.4 and clade 2.3.2.1 or anti-NS1 monoclonal antibody (sc-130568; Santa Cruz Biotechnology). Antisera were prepared by immunizing Balb/c mice with HA proteins from FJ/5 or JS/7 virus. For western blots, proteins were transferred to nitrocellulose membranes. Membranes were blocked at room temperature for 1 h in blocking buffer (150 mМ NaCl, 20 mM Tris–HCl, pH 7.5, 5% skimmed milk powder). Blots were probed with mouse anti-H5 serum at 1:2000 in blocking buffer containing 0.1% Tween-20 for 2 h at room temperature. Following three washes with TBST buffer (150 mM NaCl, 20 mM Tris–HCl, pH 7.5, 0.1% Tween-20), blots were incubated for 1–2 h at room temperature in HRP-conjugated goat-anti-mouse IgG antibody (Boster, Wuhan, China). The protein expressions were visualized by enhanced chemiluminescence system using SuperSignal West Pico (Thermo Fisher Scientific, MA, USA) (supplementary information).

### Antigenic analyses

Antigenic analyses were performed using cross-HI tests with polyclonal antisera against the indicated viruses. To generate the antisera, 21-day-old SPF chickens were injected with 1 ml of oil emulsion-inactivated vaccines derived from FJ/5 (clade 2.3.4.4 H5) and JS/7 (clade 2.3.2.1 H5), and sera were collected 3 weeks after the injection. Antibodies to HI were tested with 0.5% (vol/vol) chicken erythrocytes.

### Preparation of vaccines

Vaccine samples were prepared from viral constructs PR8-2344H5, PR8-2321H5, and PR8-H5-NS1(73)H5, accumulated in 10-day-old ECEs at 37 °C for 72 h. Formalin (final concentration, 0.1%) was added to inactivate allantoic suspensions with viral constructs before incubating at 4 °C for 72 h. Inactivated viruses were concentrated, purified through 10–50% sucrose density gradients, and resuspended in phosphate-buffered saline (PBS). Allantoic suspensions of PR8-2344H5 and PR8-2321H5 were combined into single pools at a 1:1 ratio to obtain the combination vaccine formulation. Oil-adjuvant whole-virus inactivated vaccine was prepared with viral constructs. Inactivated virus was mixed with mineral oil adjuvant at 1:2 (vol/vol) and emulsified and the HA protein content in the final vaccine preparation is about 9.24 μg/ml, which was quantified as previous described^10^.

### Vaccination and challenge test

Eighty three-week-old white Leghorn SPF chickens were randomly divided into four groups with 20 chickens in each group. Three groups were injected intramuscularly (i.m.) with 0.3 ml formalin-inactivated PR8-H5-NS1(73)H5 vaccine, combination vaccine of PR8-2344H5 and PR8-2321H5, or PR8-2344H5 vaccine, respectively. One group was injected i.m. with 0.3 ml PBS as a control. Three weeks after vaccination, serum samples were taken from all chickens for antibody detection, and birds were challenged intranasally with 10^6^ EID_50_ of lethal H5 virus in a volume of 0.2 ml. Oropharyngeal and cloacal swabs were collected on day 3 and day 5 postchallenge (p.c.) for virus isolation and titration in eggs. All birds were observed for signs of disease or death for 10 days after challenge.

### Antibody detection using the HI assay

Specific antibodies in the chicken sera were detected using the HI assay as described previously^11^. Briefly, the sera were inactivated by incubation at 56 °C for 30 min. Then, the sera were 2-fold serially diluted with PBS, and incubated with four hemagglutination units of the target influenza virus for 30 min. This was followed by adding equal volumes of fresh 1.0% (v/v) chicken red blood cells and further incubation of 30 min. The sample HI titer was defined as the reciprocal of the highest dilution that completely inhibits the agglutination.

### Virus isolation and titration

Oropharyngeal and cloacal swabs collected were suspended in 1.0 ml of storage medium (Penicillin [2000 IU/mL], Streptomycin [2 mg/mL], Amikacin [1000 IU/mL], Nystatin [2000 IU/mL], 10% glycerol [V/V] in sterile PBS [pH = 7.2])^12^ in 2.0 ml microcentrifuge tubes.The samples were centrifuged at 10000 g for 5 min at 4 °C, and then the supernatants were applied in ten-fold dilution series and the EID_50_ was determined as above.

## Results

### Generation of recombinant influenza A virus expressing H5 HA1 protein

**Figure 1:**
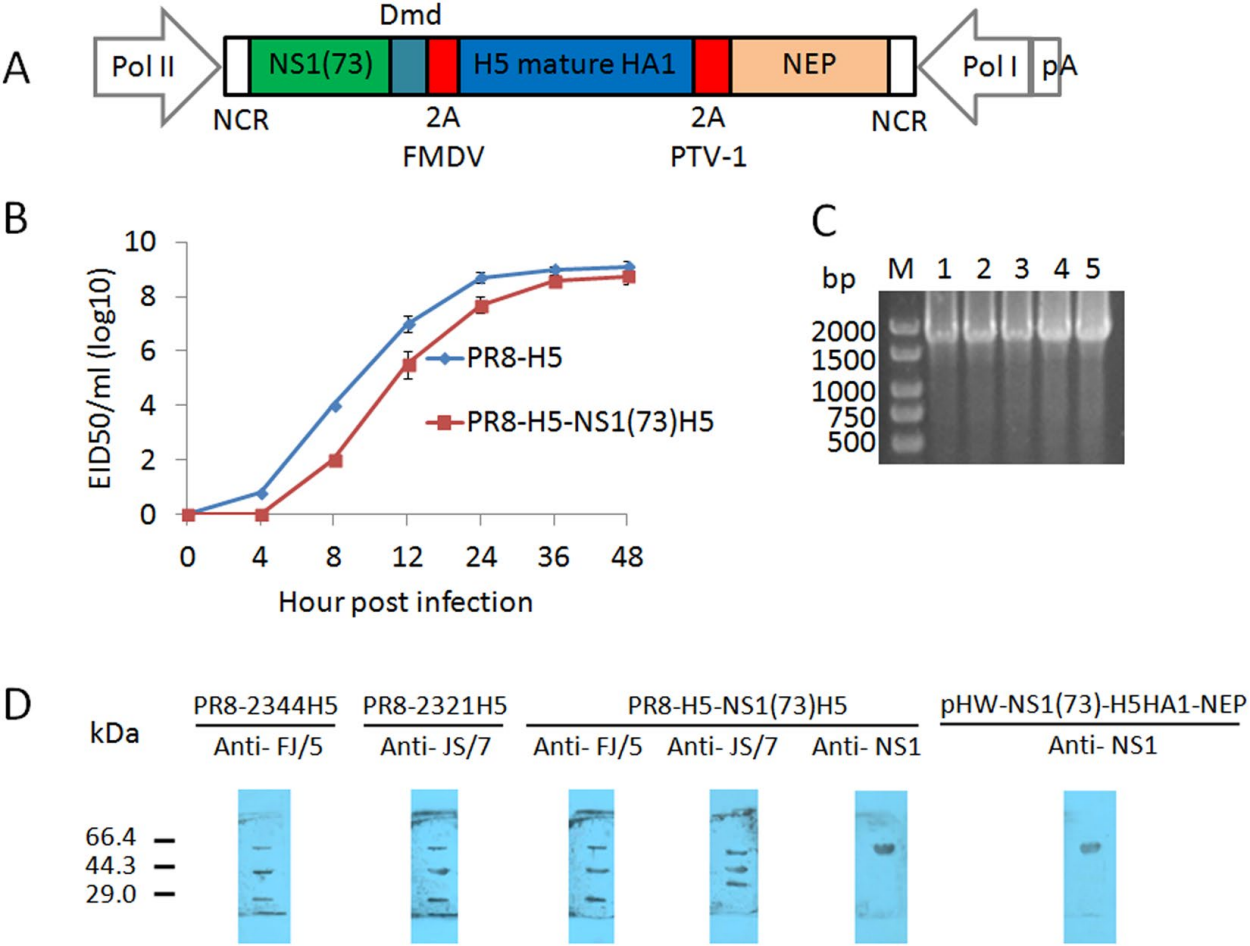
*In vitro* characterization of PR8-H5-NS1(73)H5 virus. (**A**) Schematic representation of promoters and coding sequences of pHW-NS1(73)-H5 HA1-NEP plasmid used to generate recombinant influenza virus. (**B**) Growth kinetics of PR8-H5-NS1(73)H5 and PR8-2344H5 in ECEs: 10^4^ EID_50_ per virus was inoculated into ECEs. At 4, 8, 12, 24, 36, and 48 h p.i., allantoic fluids were collected and titrated in ECEs. Shown is mean with standard error for each data point. (**C**) Genetic stability of PR8-H5-NS1(73)H5 in chicken embryos, by RT-PCR. 1) pHW plasmid encoding NS1(73)-H5 HA1-NEP genes; 2) Passage 1 of PR8-H5-NS1(73)H5; 3) Passage 3 of PR8-H5-NS1(73)H5; 4); Passage 5 of PR8-H5-NS1(73)H5; 5) Passage 10 of PR8-H5-NS1(73)H5. (**D**) ECEs infected with PR8-2344H5, PR8-2321H5, or PR8-H5-NS1(73)H5 virus with allantoic fluids purified by ultracentrifugation through a 20% sucrose cushion. Proteins were visualized by western blot using anti-H5 against a different clade or anti-NS1 monoclonal antibody.

We designed a tricistronic NS-derived gene segment with a single open reading frame comprising NS1(1–73)Dmd, H5 HA1 and NEP separated by two different 2A self-processing sites. We used different 2A peptide sequences to reduce the risk of recombination at these sites, which could lead to excision of the HA1 coding information. A foot-and-mouth disease virus (FMDV) 2A autoprocessing site was inserted between NS1(1–73)Dmd and HA1, with HA1 separated from NEP by a porcine teschovirus-1 (PTV-1) 2A cleavage site (Fig. 1A). The FMDV 2A peptide was the first 2A cleavage site to be described, and has been used for applications including the generation of recombinant influenza viruses^13–16^. The PTV-1 2A cleavage site has a high cleavage efficiency^17,18^. This artificial NS segment was used to rescue influenza viruses expressing dimeric NS1(1–73), HA1 and NEP in a PR8 virus genetic background^8^. Based on the HA and NA gene sequences of FJ/5 replacing the corresponding gene sequences of PR8 virus, this rescue was successful. We named the resulting virus PR8-H5-NS1(73)H5.

### *In vitro* characterization of the PR8-H5-NS1(73)H5 virus

To assess if truncation of the NS1 gene or insertion of HA1 affected viral fitness *in vitro*, we compared growth kinetics in ECEs with corresponding parental PR8-H5 virus. We used multicycle replication assays in ECEs to compare growth kinetics of PR8-H5-NS1(73)H5 and its parental PR8-H5 virus. We inoculated ECEs with 10^4^ EID_50_ of virus and determined viral titers in allantoic fluid at different time points after inoculation. Although the peak titer of these viruses were similar after two days (Fig. 1B; P > 0.05, paired *t*-test), PR8-H5-NS1(73)H5 did replicate slower than PR8-H5. During initial passages in ECEs, PR8-H5-NS1(73)H5 had low infection and hemagglutination titers; however, as the number of passages increased, titers also increased (Table 1). By the fifth passage, the infectious titer of PR8-H5-NS1(73)H5 was 8.83 ± 0.15 log10 EID_50_/ml.

**Table 1:** Infection and hemagglutination titers for the PR8-H5-NS1(73)H5 during passage in embryonated chicken eggs.

Passage	Infection (log10 EID_50_/ml)	Hemagglutination titer
1	6.42 ± 0.24	1:128
3	7.87 ± 0.20	1:256
5	8.83 ± 0.15	1:512

Examination of the NS1 gene by RT-PCR confirmed that PR8-H5-NS1(73)H5 retained its HA1 inserts in ECEs for more than ten passages (Fig. 1C). Sizes of the NS1 genes from viral constructs containing H5 HA1 protein corresponded to the size of genes amplified from the control pHW plasmids (1785bp). These results were confirmed by sequencing, which showed that the nucleotide sequences of the NS1 gene of PR8-H5-NS1(73)H5 corresponded to the H5 HA1 protein (data not shown).

To test if HA1 was expressed in ECEs, we inoculated ECEs with 10^4^ ELD_50_ PR8-2344H5, PR8-2321H5 or PR8-H5-NS1(73)H5 virus and performed western blots. Detection with anti-FJ/5 of PR8-H5 or PR8-H5-NS1(73)H5 virus samples revealed three major bands of approximately 63 kDa, 36 kDa and 27 kDa (Fig. 1D); these were HA0, HA1, and HA2, respectively. Detection with anti-JS/7 of PR8-2321H5 virus samples revealed three major bands of approximately 63 kDa, 36 kDa and 27 kDa (Fig. 1D), which were HA0, HA1, and HA2, respectively. Detection with anti-JS/7 of PR8-H5-NS1(73)H5 virus samples revealed three major bands of approximately 59 kDa, 46 kDa and 36 kDa (Fig. 1D). The higher band most likely corresponded to uncleaved polyprotein (predicted size of 67.4 kDa) and the band of 36 kDa to HA1. The middle band likely corresponded to NS1Dmd-HA1 (predicted size 52.9 kDa) or the HA1-NEP fusion protein (predicted size 52.2 kDa) (Fig. 1D). Detection with an anti-NS1 monoclonal antibody revealed only a band of 59 kDa in lysates of PR8-H5-NS1(73)H5 infected cells, corresponding to uncleaved precursor protein(Fig. 1D). Since the anti-NS1 antibodies did not reveal the 46 kDa band that was detectable with anti-JS/7, this band most probably corresponds to the HA1-NEP fusion protein (Fig. 1D). Western blots indicated that cleavage at the 2A cleavage sites was incomplete. PR8-H5-NS1(73)H5 virus replicated as efficiently as a corresponding parental PR8-H5 virus *in vitro*. Infection of cells with PR8-H5-NS1(73)H5 virus resulted in H5 HA1 expression, although processing at the introduced 2A sites was incomplete.

### Antigenic analyses

To determine how much antigenic diversity between these viruses, we examined the antigenicity by cross HI assay. The results showed that FJ/5 (clade 2.3.4.4 H5) isolate exhibited weak reactivity with JS/7 (clade 2.3.2.1 H5) antisera (Table 2). The cross-reactive HI titers of FJ/5 antiserum against JS/7 isolate were as much as 8 Log2 lower than that against the homologous FJ/5 antigen (9 Log2). In contrast, the cross-reactive HI titers of the JS/7 antiserum against FJ/5 antigen were as much as 8 Log2 lower than that of homologous JS/7 isolate (9 Log2).

**Table 2:** Results of HI assays using FJ/5 and JS/7 antiserum for JS/7 and FJ/5 isolates*.

Isolate	Antibody titer, log2	
	FJ/5	JS/7
FJ/5	**9**	1
JS/7	1	**9**

^*^FJ/5 and JS/7 antiserum were generated by vaccinating specific-pathogen free chickens with the oil-emulsified inactivated A/chicken/Fujian/5/2016(H5N6) (FJ/5, clade 2.3.4.4) and A/chicken/Jiangsu/7/2017(H5N1) (JS/7, clade 2.3.2.1) vaccines, respectively. Hemagglutination inhibition titers against the homologous antigen/virus are shown in boldface.

### Protective efficacy of PR8-H5-NS1(73)H5 vaccine

The PR8-H5-NS1(73)H5 vaccine was highly immunogenic in chickens. Three weeks after a single vaccination, mean hemagglutinin inhibition (HI) antibody titers to the homologous clade 2.3.4.4 H5 virus were 8.6 ± 0.6 log2 (Table 3). HI antibody titers induced by PR8-H5-NS1(73)H5 vaccine to clade 2.3.4.4 H5 virus were comparable for titers from the combination vaccine of PR8-2344H5 and PR8-2321H5.

**Table 3:** Efficacy of vaccines against clade 2.3.4.4 and clade 2.3.2.1 H5 highly pathogenic avian influenza viruses in chickens.

Vaccine group	Challenge virus	Mean HI titer 21 days after immunization (log2)	Virus shedding (EID_50_/ml)				Survival
			Day 3 p.c.^a^		Day 5 p.c.		
			Oropharyngeal	cloacal	Oropharyngeal	cloacal	
PR8-H5-NS1(73)H5	FJ/5	8.6 ± 0.6	0/10	0/10	0/10	0/10	10/10
	JS/7	5.4 ± 0.5	0/10	0/10	0/10	0/10	10/10
PR8-2344H5 + PR8-2321H5	FJ/5	8.8 ± 0.9	0/10	0/10	0/10	0/10	10/10
	JS/7	8.4 ± 0.5	0/10	0/10	0/10	0/10	10/10
PR8-2344H5	FJ/5	8.9 ± 0.4	0/10	0/10	0/10	0/10	10/10
	JS/7	0.8 ± 0.4	4/4 (4.2 ± 0.5)	4/4 (4.4 ± 0.4)	NA^b^	NA	0/10
Control	FJ/5	<1	4/4 (4.5 ± 0.6)	4/4 (4.8 ± 0.5)	NA	NA	0/10
	JS/7	<1	4/4 (4.1 ± 0.3)	4/4 (4.5 ± 0.4)	NA	NA	0/10

^a^pc: post challenge.

^b^NA: not applicable due to death of chickens.

Mean HI antibody titers induced by PR8-H5-NS1(73)H5 vaccine to the homologous clade 2.3.2.1 H5 virus reached 5.4 ± 0.5 log2 (Table 3). HI antibody titers induced by PR8-H5-NS1(73)H5 vaccine to clade 2.3.2.1 H5 virus were significantly lower than titers from the combination vaccine of PR8-2344H5 and PR8-2321H5 (*P* < 0.01, paired *t*-test).

Oropharyngeal and cloacal swabs were collected from all chickens on days 3 and 5 p.c. to assess virus shedding. All vaccinated chickens remained healthy after challenge with homologous clade 2.3.4.4 and clade 2.3.2.1 H5 HPAIVs (Table 3). Virus shedding was not detected from any bird on days 3 or 5 p.c.; however, all control birds shed viruses and died within 4 days p.c. with clade 2.3.4.4 and clade 2.3.1 H5 HPAIVs (Table 3). Moreover, univalent PR8-2344H5 vaccine group challenged with FJ/5 remained healthy and no virus shedding was detected; however, when challenged with JS/7, all birds shed viruses and died within 4 days p.c. (Table 3).

## Discussion

H5N1 AIVs have become enzootic in poultry and wild birds in China^2^. The government of China undertook a mass poultry vaccination practice against HPAI in 2005^19^. Vaccine strains used in China have been updated several times since 2004 to ensure an antigenic match between the vaccines and the prevalent strains^5^. Currently, combination vaccine of H5 Re-8 and H7 Re1 has been used to control highly pathogenic H5 and H7N9 AIVs throughout the country since August 2017. However, clade 2.3.2.1 H5 virus is also a threat for poultry, with some outbreaks caused by clade 2.3.2.1 H5 virus.

In this study, we generated a replication-competent recombinant influenza A virus of subtype Н5N1 expressing another H5 HA1 protein. We demonstrated that this vaccine could be used as a new candidate for H5 subtype avian influenza vaccines against two clades. Current killed influenza virus vaccines predominantly induce anti-HA that specifically targets antigenic sites in the globular head domain of the HA1 region and block receptor binding^20,21^. Therefore, we chose mature HA1 as an antigenic determinant inserted into the NS segment between NS1 and NEP. Our strategy resembles that of De Baets *et al*., who reported inserting GFP into an NS1 segment of PR8 virus^22^. We adapted this strategy to generate a virus expressing both HA and NA of H5N6 and HA1 of H5N1. To avoid expression of an NS1-fusion protein, we designed a tricistronic NS segment containing NS1(1–73)Dmd, H5 HA1 and NEP separated by different 2A auto-cleavage sites. Although this strategy should theoretically result in three separate proteins, western blots of infected ECEs showed that cleavage was only partial. Evaluating the cleavage efficiency of the two 2A sites could be interesting. Polyprotein processing could be improved by changing or optimizing one or both of the 2A autoproteolytic cleavage sites, as not all 2A sites show the same cleavage efficiency^16^. Nonetheless, the efficiency of different 2A sites must be determined empirically, as they may depend on context. To reduce the NS segment size, we used a truncated NS1 protein that generally results in *in vivo* virus attenuation^23^. Some attenuation can be reversed by adding a heterologous dimerization domain to the truncated NS1, which improves stability of the dimeric NS1 protein^24^.

Foreign gene-expressing viruses ideally have high genetic stability. This is difficult to accomplish with engineered influenza viruses. PR8-H5-NS1(73)H5 virus has *in vitro* replication kinetics that are similar to parental PR8-H5 virus, although PR8-H5-NS1(73)H5 replicated slower than PR8-H5. Sequencing showed that there was no mutation in the third, fifth and tenth passage of PR8-H5-NS1(73)H5 virus stock compared to the pHW plasmid encoding the corresponding gene. This result indicated the stability of the PR8-H5-NS1(73)H5 virus. The reason for the virus grows better after several passages needs further investigation. A previous study demonstrated that NS1 tolerates foreign sequences exceeding its own length^7^. As far as we know, GFP is the longest fragment inserted, at 702 bp. We have successfully inserted 978 bp of H5 mature HA1 and obtained stable virus.

As compared with FJ/5 (clade 2.3.4.4 H5) and S/7 (clade 2.3.2.1 H5), the amino acid identity of HA was 90.5%, suggesting that these viruses are likely distinct in antigenicity. This is consistent with the results of the cross-HI tests and the vaccination-challenge tests.

Upon vaccination of SPF chickens with inactivated vaccine PR8-H5-NS1(73)H5, specific HI antibody responses were induced. At 21 days post vaccination, HI antibody titers against clade 2.3.4.4 H5 induced by PR8-H5-NS1(73)H5 vaccine were comparable to titers from the combination vaccine of PR8-2344H5 and PR8-2321H5. However, HI antibody titers against clade 2.3.2.1 H5 by PR8-H5-NS1(73)H5 vaccine were significantly lower than titers from the combination vaccine of PR8-2344H5 and PR8-2321H5 (*P* < 0.01, paired *t*-test). In spite of this result, inactivated vaccine PR8-H5-NS1(73)H5 provided complete protection against both H5 HPAIV of clade 2.3.4.4 and clade 2.3.2.1 challenge.

In conclusion, we generated a bivalent vaccine candidate, PR8-H5-NS1(73)H5, expressing both clade 2.3.4.4 H5N6 HA and clade 2.3.2.1 H5N1 HA1 of AIVs. To our knowledge, this is the first report of one kind of influenza virus simultaneously expressing two proteins from AIVs in different clades. This vaccine candidate induced HI antibody titers against both clade 2.3.4.4 and clade 2.3.2.1 H5 antigens, although titers against clade 2.3.2.1 H5 were significantly lower than from the combination vaccine of PR8-2344H5 and PR8-2321H5 (*P* < 0.01, paired *t*-test). PR8-H5-NS1(73)H5 provided complete protection against both clades of HPAI H5N6 and HPAI H5N1 in challenges. Our results indicated that PR8-H5-NS1(73)H5 was highly immunogenic in chickens.

## Electronic supplementary material

Supplementary information
