# Supplementary material for: Influenza viral vectors expressing two kinds of HA proteins for bivalent vaccines against clade 2.3.4.4 and clade 2.3.2.1 H5 HPAIVs
Source: Sci Rep. 2018 Jun 19;8:9327. doi: 10.1038/s41598-018-27722-5 (PMC6008415; doi:10.1038/s41598-018-27722-5)
Supplement: Supplementary file 1 — Supplementary information [file 41598_2018_27722_MOESM1_ESM.pdf]

Influenza viral vectors expressing two kinds of HA proteins for bivalent vaccines against clade 2.3.4.4 and clade 2.3.2.1 H5 HPAIVs

Guangyu Hou, Jinping Li, Yan Wang, Suchun Wang, Cheng Peng, Xiaohui Yu, Jihui Jin, Wenming Jiang

Supplementary Figure

Supplementary Figure S1

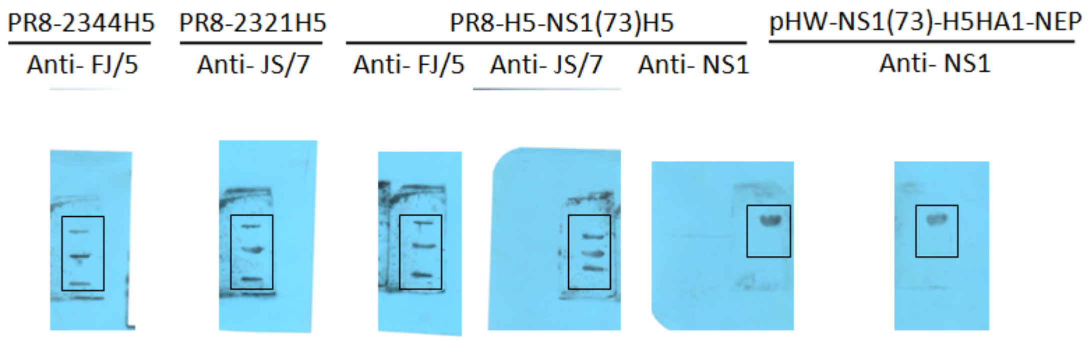

Figure S1 Uncropped western blot used in Fig 1D
